# Methylene blue and methyl orange removal using green rust as a low-cost, sustainable adsorbent and photocatalyst†

**DOI:** 10.1039/d5ra01508a

**Published:** 2025-06-02

**Authors:** Nosaiba M. El Kawas, Ayman H. Zaki, Mohamed Taha

**Affiliations:** a Materials Science and Nanotechnology Department, Faculty of Postgraduate Studies for Advanced Sciences (PSAS), Beni-Suef University Beni-Suef Egypt ayman.zaki@psas.bsu.edu.eg

## Abstract

Green rust (GR), a mixed-valent iron mineral from the layered double hydroxides family, has gained attention for its potential in environmental and energy applications. Despite its widespread presence and possible role in life's evolution, GR's susceptibility to oxidation has limited its practical use. In this study, we prepared a stabilized GR, synthesized through a one-pot solvothermal method using iron(iii) chloride and glycerol. X-ray diffraction, field emission scanning electron microscopy, Fourier transform infrared spectroscopy, and thermogravimetric analysis characterized the prepared green rust. The adsorption of the anionic dye methyl orange (MO) and its mixture with the cationic dye methylene blue (MB) onto GR was explored for the first time. Various parameters, such as pH (5–11), adsorbent dose (0.015–0.030 g), and initial dye concentration (10–200 mg L^−1^), were studied to determine the efficiency of GR for removing MO from water. To get insights into the adsorption mechanism and kinetics, different isotherms and kinetic models were applied to fit the data of adsorption obtained at pH 7. The obtained data revealed that the adsorption process obeys pseudo-first-order, pseudo-second-order, mixed 1,2-order, and Avrami models. The isotherm data showed strong agreement with the Langmuir–Freundlich, Sips, and Baudu models. At 25 °C, the maximum adsorption capacity for MO was 93.18 mg g^−1^. Monte Carlo and molecular dynamics simulations were used to identify specific binding sites, quantify adsorption energies, and elucidate the mechanisms driving the removal process.

## Abstract 2

Green rust (GR), a mixed-valent iron mineral from the layered double hydroxides family, has gained attention for its potential in environmental and energy applications.

## Graphical abstract

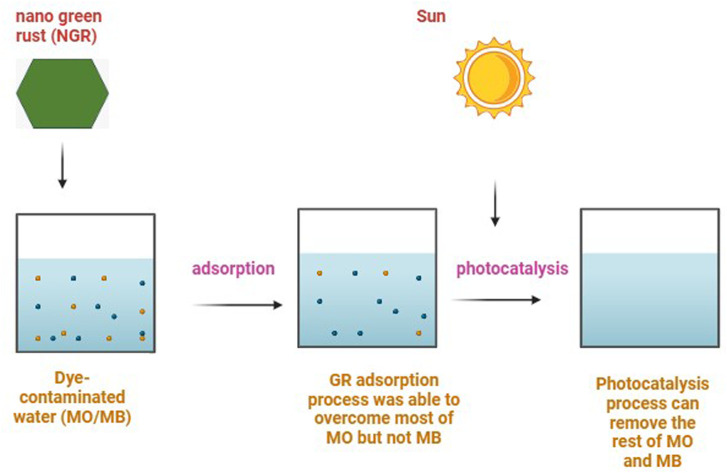

## 1. Introduction

In recent years, environmental worries about the prevalence of synthetic dyes in water bodies have grown, poisoning substantial threats to ecosystems and human health.^1^ These dyes, which are commonly found in industrial effluents from the textile, leather, and dyeing sectors, remain in water sources due to their complicated chemical structures and resistance to conventional treatment procedures.^2,3^ A significant quantity of synthetic dyes is currently utilized in industries such as textiles, leather, cosmetics, food, printing, and paper processing.^4,5^ Unfortunately, 10–15% of these dyes end up in industrial wastewater, making them a major source of environmental pollution.^6^ Many of these dyes are harmful to aquatic plants and animals, and some can even be carcinogenic to humans like methyl orange (MO) and methylene blue (MB).^7^

Methyl orange is a very common anionic azo-dye that is used in many industries and applications.^8^ When entering the body it will be a substrate for the reductive enzymes in the liver, and subsequently a carcinogenic aromatic amines will be produced.^9,10^

Methylene blue is a common cationic dye used as a biological stain, a dye in the textile industry, and in various other applications.^11–14^ Despite its low concentration (<1 mg L^−1^), MB has an extremely high color intensity and is considered a hazardous pollutant.^15^ To save humans and other livings' lives, researchers developed many methods and materials to treat polluted water before the discharge into the environment.^16–20^

To reduce the concentration of dyes in industrial wastewater before it’s released into the environment, various treatment methods have been proposed novel ways that are both practical and ecologically friendly, including coagulation, flocculation, adsorption, ion exchange, oxidation, chemical precipitation, ultrafiltration, catalytic ozonation, biodegradation, and electrocoagulation.^21^ However, the complex chemical structure of dyes and their resistance to biodegradation make traditional degradation techniques less effective, potentially leading to byproducts with even higher toxicity.^21^ Consequently, adsorption is considered a more suitable method for dye removal, as it is a highly versatile water treatment process with low operating costs, a simple design, and effectiveness at extremely low concentrations.^21^

Various adsorbents are utilized to remove these dyes, including polymers, clays, metal oxides, layered double hydroxides (LDHs), and others.^22–28^ LDHs have emerged as effective adsorbents for removing MO and MB from water due to their tunable structure, high surface area, and anion-exchange capabilities. For instance, Mg–Al LDHs have been used to remove MO,^22^ while nickel-modified Mg–Al LDHs (Ni/Mg/Al-LDHs) further enhance MO removal efficiency.^23^ For MB, various LDH-based materials have been employed, including Mg–Al LDH/biochar composites,^24^ Zn–Al LDHs modified with plant extracts (Zn–Al LDH@ext),^25^ calcined Mg/Al/Fe LDHs,^26^ and Co–Fe LDHs.^27^ Additionally, Zn–Fe LDHs have been utilized for the adsorption of both MO and MB dyes.^28^ Among the LDH materials, green rust (GR) is notable. One intriguing option gaining traction is the use of GR, a class of iron-based compounds renowned for their unique redox characteristics and capacity to interact with organic contaminants. Green rusts, with their layered structure and strong reactivity to pollutants, provide a promising option for dye removal from aquatic settings. Their composition and surface chemistry make them ideal candidates for adsorption and the subsequent breakdown of dye molecules, reducing water pollution.

Green rust (GR), a type of LDH material, is a promising pollutant removal agent due to its unique redox properties, strong interactions with organic contaminants, and high reactivity. Its layered structure and surface chemistry enable both adsorption and degradation of pollutants, making it effective for water purification. Previous studies have demonstrated the efficacy of GR in removing various pollutants, including heavy metals such as vanadium(v) and chromium(vi), with adsorption capacities of 30.66 mg g^−1^ and 39.48 mg g^−1^, respectively.^29^ Additionally, GR has been utilized in the catalytic degradation of tribromophenol when doped with palladium(ii).^30^

This study explores the feasibility and effectiveness of using GR for the removal of MO and MB from water. By analyzing adsorption processes, kinetics, and isotherms, it seeks to optimize conditions for efficient dye removal, offering a cost-effective and eco-friendly water treatment solution. The research is significant for environmental remediation, as it leverages GR's capabilities to address dye pollution and enhance water quality. Ultimately, this work aims to advance sustainable water management and contribute to a healthier aquatic ecosystem.

## 2. Materials and methods

### 2.1. Preparation of GR

**Fig. 1:**
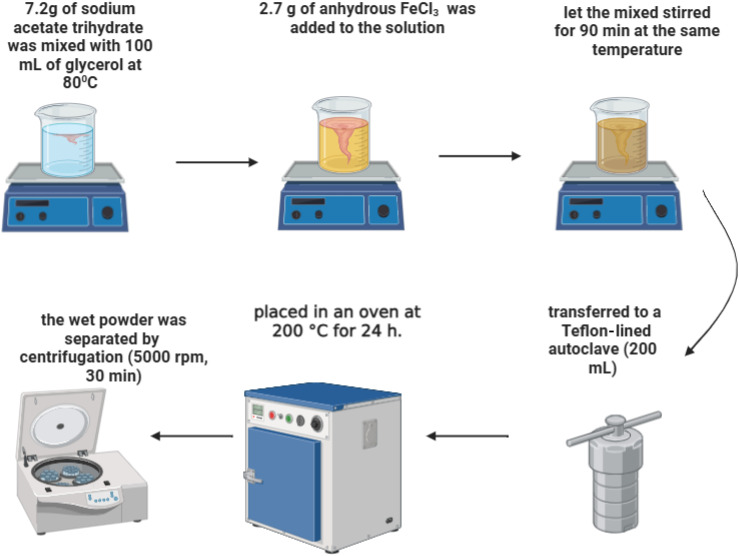
Schematic representation of the GR preparation process.

2.7 g of the anhydrous form of FeCl_3_ (Alpha Chemika, ≥97.0%) and were dissolved in 100 mL of glycerol (Sigma Aldrich, 99.5%) containing 7.2 g of sodium acetate trihydrate (7.2 g, 0.05 mol, LOBA CHEMIE, ≥99.0%), heated at 80 °C. The mixture was stirred for 1.5 h under the same conditions. The solution was then transferred to a 200 mL Teflon-lined autoclave, sealed, and heated in an oven at 200 °C for 24 hours. The autoclave was left to cool-down till reaching room temperature, the resulting wet powder was collected by centrifugation (5000 rpm for 30 minutes), washed twice with ethanol (≥99.8%), followed by two washes with a 50 : 50 ethanol–water mixture, and then rewashed with pure ethanol,^31^ as illustrated in Fig. 1. Finally, the sample was dried at 80 °C for 12 hours.

### 2.2. Characterizations

Powder X-ray diffraction (XRD) patterns were recorded by Panalytical Empyrean 3, Malvern, Netherlands, with Cu target at 40 kV and 40 mA, scan rate of 5° min^−1^. The Fourier-transform-infrared-spectrometer (FTIR) spectra of the GR were obtained with Bruker-Vertex 70 performed. Field-emission scanning electron microscope (SEM) images were taken on ThermoFisher (USA) Quattro S Felid Emission Gun, Environmental SEM “FEG ESEM”. Setaram Labsys Evo S60 analyzer was used to perform thermogravimetric analysis (TGA) for the prepared green rust, with a heating rate of 10 °C min^−1^ till reaching 900 °C. The Shimadzu UV-vis spectrophotometer 1800 was used to determine the MO and MB concentrations at their respective *λ*_max_ values (465 nm and 663 nm, respectively).

### 2.3. Adsorption process

The adsorption of MO onto the surface of GR was examined under various conditions, including pH, adsorbent dosage, initial dye concentration, and contact time. A 1000 mg per L stock solution of both MO was prepared. To assess the effect of pH, 25 mL of 100 mg per L MO was mixed with 0.3 g of GR at pH values of 5.0, 7.0, 9.0, and 11.0. The pH was adjusted using either 0.1 M HCl or 0.1 M NaOH. Each experiment was conducted in triplicate to ensure reproducibility.

Experiments were performed with 25 mL of 150 mg per L MO using various amounts of GR (ranging from 0.01 g to 0.09 g) to investigate the effect of GR dosage. Additionally, the influence of initial dye concentrations (ranging from 10 to 350 mg L^−1^) on adsorption was studied using the optimal GR dosage of 0.03 g and its corresponding optimal pH of 7.0.

All samples were shaken overnight to ensure proper adsorption.1
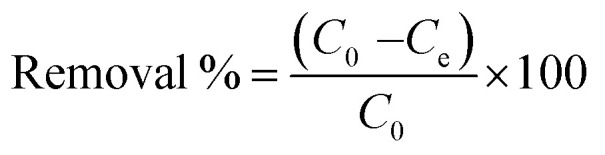
where *C*_0_ = initial dye concentration and *C*_e_ = the remaining dye concentration after adsorption.

### 2.4. Isothermal models

Equilibrium isotherm data, reflecting the impact of dye concentration, were analyzed using several models, including the Freundlich, Langmuir, Langmuir–Freundlich, Sips, and Baudu isotherms, with their respective equations provided in the ESI.† The Freundlich isotherm is an empirical model that assumes a heterogeneous adsorbent surface with varying adsorption energies at different sites, allowing for multilayer adsorption.^32^ In contrast, the Langmuir isotherm describes adsorption on a homogeneous surface, where a monolayer of adsorbate forms, assuming uniform adsorption sites and no adsorbate–adsorbate interactions.^33^ The Langmuir–Freundlich isotherm integrates features of both models, providing a more adaptable fit for experimental data.^34^ Similarly, the Sips isotherm also merges Langmuir and Freundlich characteristics, accommodating heterogeneous or homogeneous surfaces and monolayer or multilayer adsorption while predicting saturation at high concentrations.^35^ The Baudu isotherm was developed to improve the accuracy of Langmuir constant estimation by reducing discrepancies across a broader concentration range.^36^

### 2.5. Kinetic models

To study the kinetics of dye adsorption by GR, we examined pseudo-first-order, pseudo-second-order, mixed-1,2-order, and Avrami kinetics, with their corresponding equations detailed in the ESI.†^37,38^ The pseudo-first-order model (PFO) is a straightforward and widely utilized method for describing the kinetics of adsorption processes. This model assumes that the adsorption rate is directly proportional to the concentration of unadsorbed molecules remaining in the solution.^39^ The pseudo-second-order model (PSO) offers an alternative approach for describing the rate of adsorption, often providing a more accurate representation than the PFO. Unlike its first-order counterpart, the PSO considers the interaction between the adsorbate molecules and the available adsorption sites.^40^ The mixed-1,2-order (MO) model accounts for the complexity of adsorption by incorporating both the dependence on unoccupied sites, as seen in the PFO model, and the interactions between adsorbed and free molecules, similar to the PSO model.^41^ The Avrami model^42^ describes the overall change in the fraction of occupied adsorption sites over time. It is based on the theory of nucleation and growth, assuming absorption occurs through the process of random nucleation and growth of adsorbate on the surface.

### 2.6. Computational study

The Monte Carlo (MC) and molecular dynamics (MD) simulations were carried out by the BIOVIA Materials Studio 2020 package [https://www.3ds.com]. The structure of green rust was taken from the reference (https://www.crystallography.net/cod/9013993.html) by replacing the sulfate group (SO_4_^2−^) with the lactate group.^43^ The Bravais lattice is centered triclinic with lattice parameters, angles (106.0°, 78.3°, 117.7°), and lengths (5.056, 5.126, and 9.882) Å. The crystal structure was optimized using the GGA/PBE/OTFG-ultrasoft potential with TS correction (energy cutoff: 571 eV) method. These calculations were performed within the CASTEP quantum mechanics module. The MO was optimized by using the (DFT/B3LYP) method with TS correction and the DNP+ basis set, as implemented in the DMOL^3^ module. Two surfaces were investigated: the (001) surface, used to elucidate the interaction between the hydroxyl (OH) groups of GR and the dye molecules, and the (010) surface, employed to reveal the interaction between the Fe cations and the dye molecules. 6U and 6V surfaces were constructed from the GR unit cell, with a 30 Å vacuum layer introduced above the surfaces. Both GR and MO were then optimized using the COMPASS III forcefield, and their Mulliken charges were utilized. The optimization process was carried out using the Forcite module with the following convergence parameters: energy at 2.0 × 10^−5^ kcal mol^−1^, force at 0.001 kcal mol^−1^ Å^−1^, and displacement at 1.0 × 10^−5^ Å. Adsorption studies for single molecule, monolayer, and multilayer configurations were performed using the Adsorption Locator tool. MC simulations were conducted with simulated annealing parameters set to 5 cycles, 50 000 steps per cycle, a maximum temperature of 1.0 × 10^5^ K, and a final temperature of 100 K. The resulting MC structures for monolayer and multilayer systems were hydrated using the Adsorption Locator tool. MD simulations were employed using the *NVT* ensemble with a time step of 1 fs, a total of 5 000 000 steps, and a temperature of 25 °C, controlled by the Nose thermostat using the specified forcefield and charges.

### 2.7. Adsorption and photocatalysis for MO and MB removal

To investigate the adsorption behavior of GR toward mixed anionic and cationic dyes, we prepared a dye mixture containing 0.01 g of GR with equal parts of an anionic dye (MO, 30 mg L^−1^, pH 7) and a cationic dye (MB, 30 mg L^−1^, pH 7). The adsorption experiment was conducted under agitation, with varying dye ratios: 50% MO: 50% MB (12.5 mL of MO, 12.5 mL of MB), 75% MO: 25% MB (45 mL of MO, 15 mL of MB), and 25% MO: 75% MB (15 mL of MO, 45 mL of MB). Samples were collected at 15, 30, 45, and 60 minutes for adsorption. The procedure was then repeated for the photocatalysis process, upon UV irradiation, with samples taken at extended time intervals (70, 80, 90, 100, and 110 minutes) while the mixture continued interacting with GR.

## 3. Results and discussion

### 3.1. Material characterization studies

#### 3.1.1. XRD analysis

**Fig. 2:**
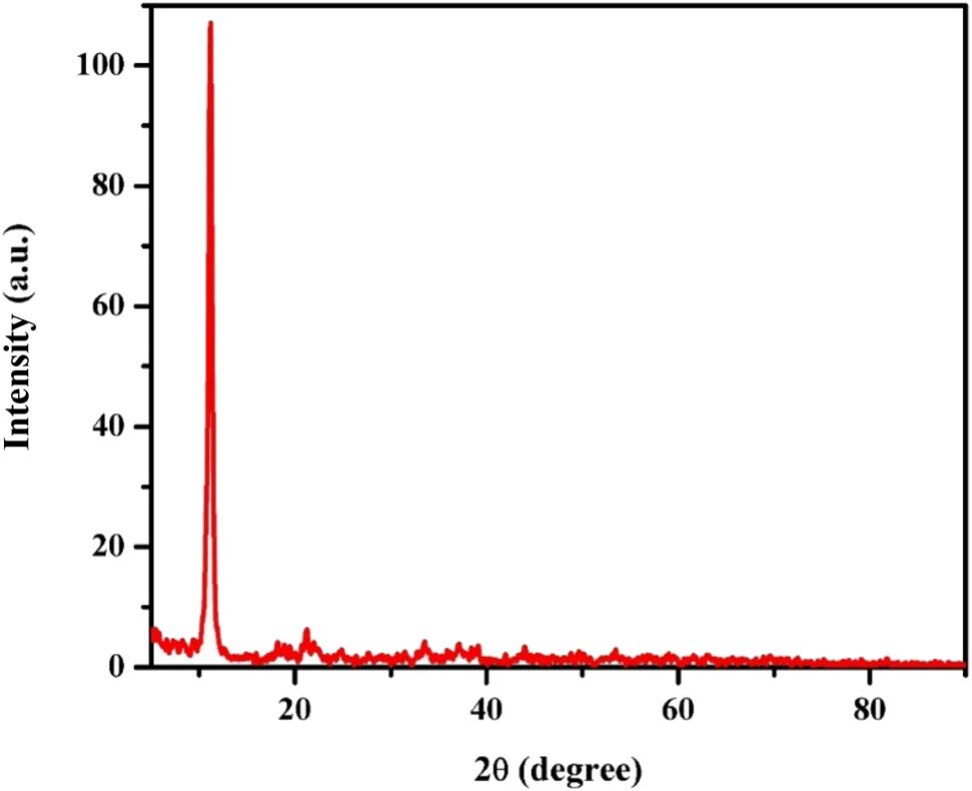
The XRD pattern of GR.

Fig. 2 presents the XRD pattern of the synthesized GR sample, which closely resembles the patterns of conventional lactate-type GR (*e.g.*, JCPDS no. 159700). The prepared GR contained lactate anions in its interlayer space. The *d* spacing of this GR sample was found to be 0.80 nm, aligning with the 0.80 nm spacing observed in other studies.

#### 3.1.2. Scanning electron microscopy

**Fig. 3:**
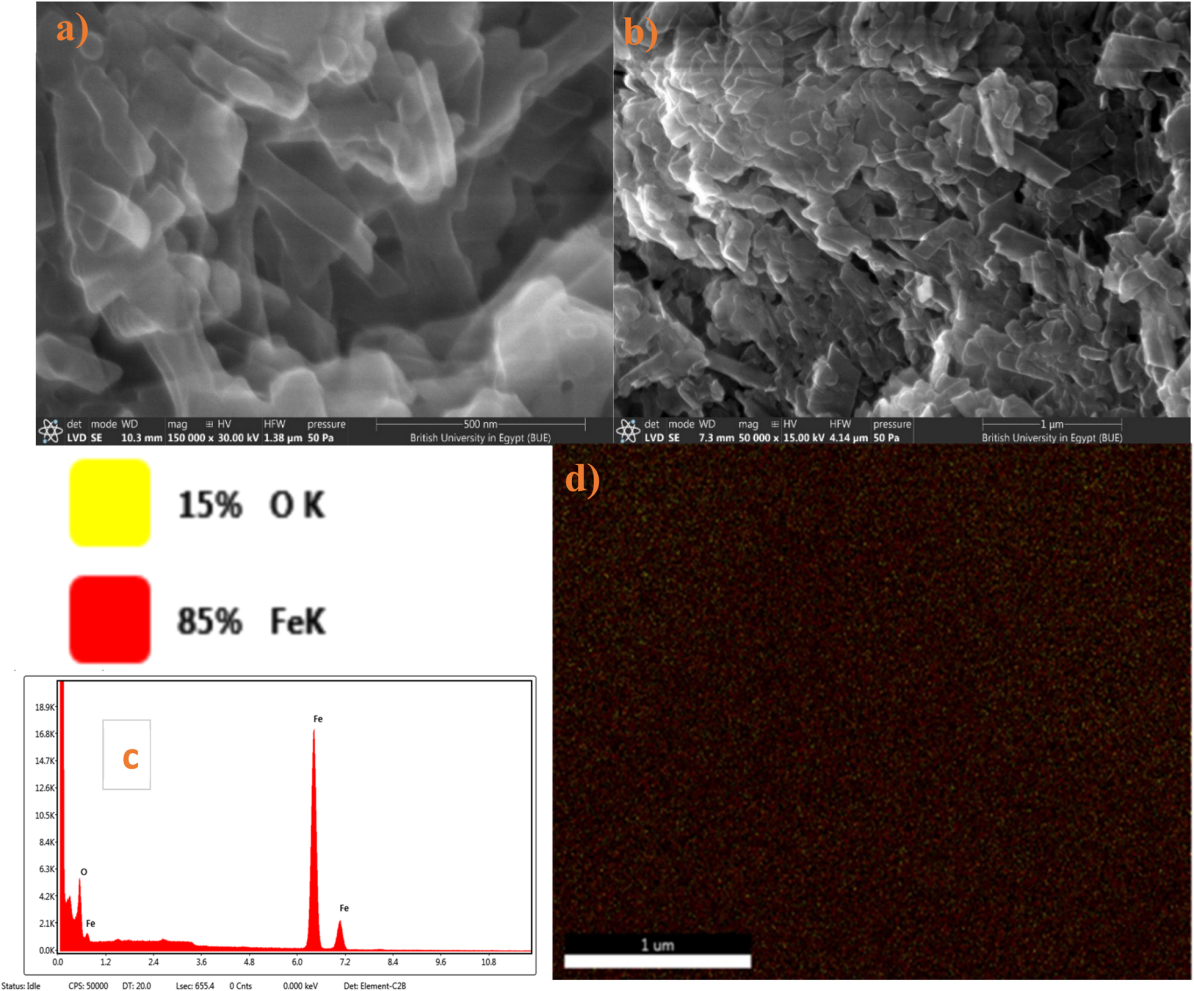
(a and b) SEM (c) EDX and (d) mapping of GR sample.

Fig. 3 shows SEM images of the GR sample, revealing particles with both belt-like and plate-like shapes. Each belt-like particle has a distinct surface texture. While the exact formation mechanism of this GR is not entirely understood. During the preparation process some of the Fe^III^ cations were reduced to Fe^II^ with unclear mechanism, the presence of the two valencies is necessary to form the iron-only LDH, taking the common morphology as shown in the FESEM images. In addition, it was proposed that the presence of lactate and carbonate anions between the green rust layers maybe attributed to glycerol decomposition.

#### 3.1.3. FTIR spectra

**Fig. 4:**
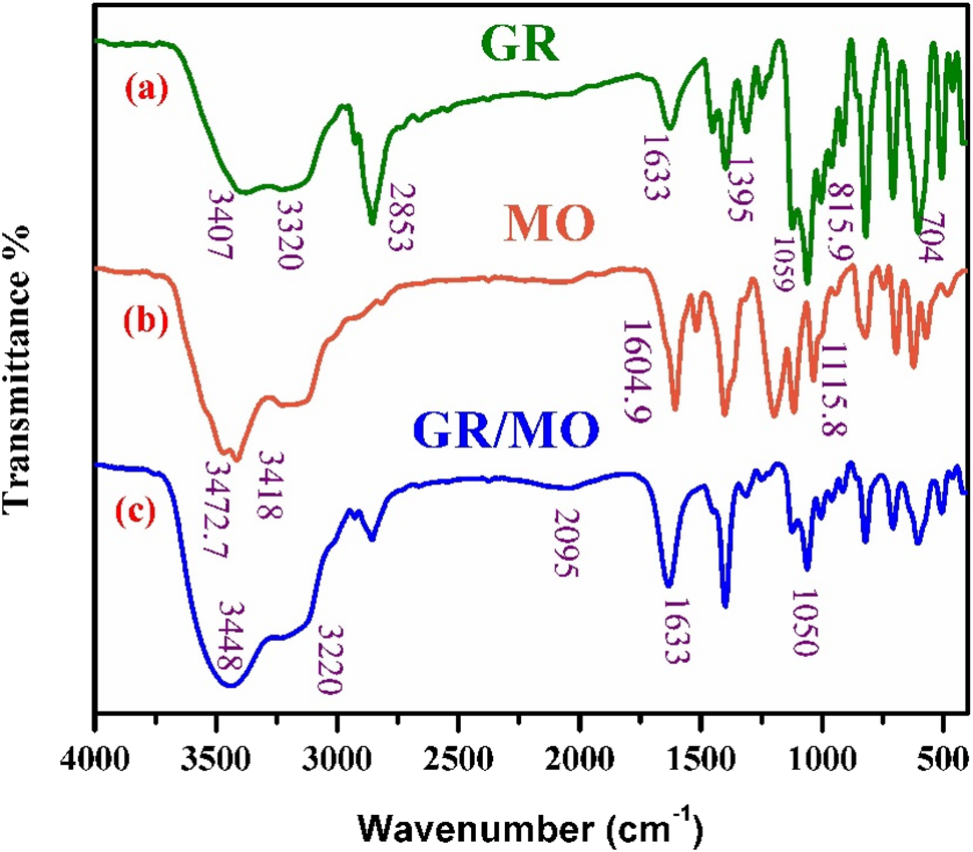
FT-IR spectra of MO and GR before and after adsorption.

Fig. 4(a) shows the FTIR spectra of GR, revealing absorption bands associated with the lactate anion (including those from carboxylate and methyl groups) as well as the carbonate anion, confirming their presence in the GR sample.^44^ The broadband around 3407 cm^−1^ is attributed to the O–H stretching vibration of GR,^45^ which suggests the presence of Fe(OH)_2_ and Fe(OH)_3_ on the surface. The peak near 1633 cm^−1^ corresponds to the C=O stretching vibration of the carbonyl group.^45^ The characteristic peak at 2853 cm^−1^ is associated with the C–H stretching vibration of aliphatic carbon.^46^ Peaks at 1059 cm^−1^, 815.9 cm^−1^, and 704 cm^−1^ in GR are attributed to C–C skeletal vibrations,^47^ likely resulting from the lactate present in the GR. Fig. 4(b) shows the FTIR spectrum of MO, with characteristic peaks at 1604.9 cm^−1^, corresponding to the aromatic ring, and 1115.8 cm^−1^, which is linked to sulfate ions in MO.^48^ The peak at 2059 cm^−1^ is associated with C–N stretching vibrations in MO.^47^Fig. 4(c) shows the FTIR spectrum of GR after adsorption of MO, where peaks at 1050 cm^−1^ and 2059 cm^−1^ are attributed to the C–N stretching vibrations of MO, confirming that MO is adsorbed on the surface of GR.^31^

#### 3.1.4. Thermogravimetric analysis

**Fig. 5:**
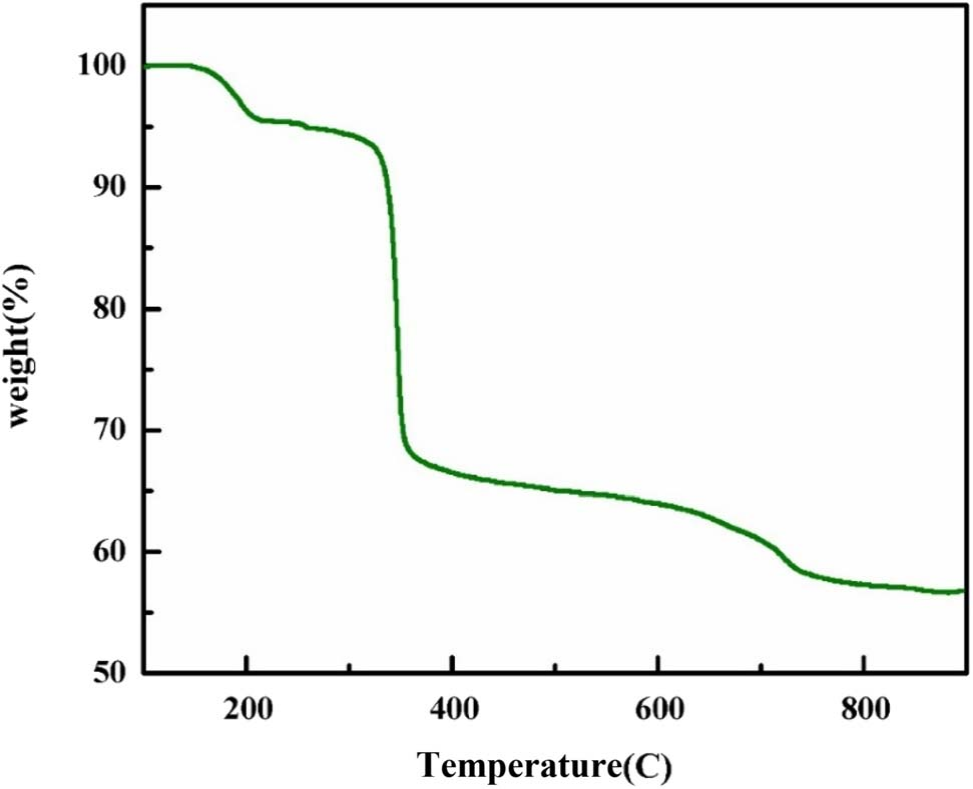
The TGA curve of the GR.

Fig. 5 presents the TGA thermogram of GR, measured from room temperature up to 900 °C at a heating rate of 10 °C per minute. The TG curve of GR reveals three primary temperature ranges associated with weight loss. The first weight reduction of approximately 4.5% corresponds to the dehydration process (theoretical value 4.5%), indicating the complete loss of water molecules from 100 °C to 217 °C. This is followed by an extended section of the curve showing a gradual weight reduction (2%) from 217 °C to 317 °C, then a sharp weight drop (25.5%) from 317 °C to 407 °C. Notably, the total weight loss is attributed to the destruction of expected compounds such as Fe(OH)_2_, Fe(OH)_3_, and carbon. The final temperature range, above 600 °C, shows a slow weight decrease 2% with a curve inflection at 670 °C. The total mass loss amounts to 43.4%.^49^

### 3.2. Adsorption process

#### 3.2.1. Effect of initial pH

**Fig. 6:**
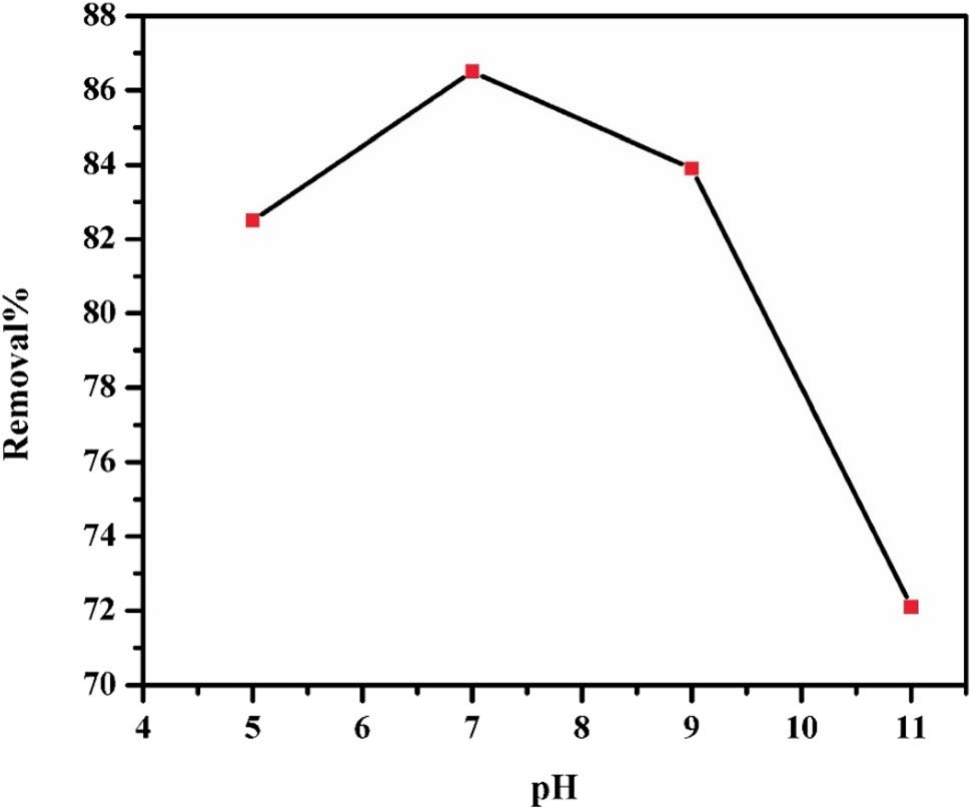
Effect of pH on the adsorption of 25 mL of 100 mg per L MO by 0.3 g of GR at 25 °C.

Studying the effect of pH change on the adsorption process is an important factor, since changing the solution pH in many cases changes the adsorption capacity. The effect of pH on MO adsorption by GR is illustrated in Fig. 6. The highest removal efficiency (%) for MO was observed at pH 7.0. Since MO is an anionic dye with negatively charged functional groups, its adsorption efficiency decreases as the pH increases. Several factors, including surface charge and the availability of active sites, influence adsorption behavior at different pH levels. The surface of LDHs contains numerous active sites that are essential for dye uptake, alongside the chemical properties of the dye in solution. The hydroxyl groups of LDH play a crucial role in adsorption, shifting between protonated (–OH_2_^+^), neutral (–OH), and deprotonated (–O^−^) states depending on pH. This impacts electrostatic interactions and, consequently, the adsorption efficiency of different pollutants. When the pH is lower than the point of zero charge (PZC) of the adsorbent, the surface becomes positively charged due to the presence of –OH_2_^+^ groups, enhancing the adsorption of negatively charged dye anions through electrostatic attraction. Conversely, at pH values above the PZC, the surface becomes negatively charged due to the formation of –O^−^, which favors the adsorption of cationic dyes.^50^ The PZC of GR was determined to be 8.3.^51^ Dye removal efficiency increased between pH 5 and 7 but declined beyond this range due to the development of repulsive forces between the negatively charged dye molecules and the adsorbent surface, reducing adsorption efficiency. The maximum adsorption efficiency observed at pH 7.0 is attributed to the synergistic effect of hydrogen bonding between the hydroxyl groups (–OH) of GR and sulphonic group of the MO, along with electrostatic attraction, which together enhance the adsorption process.

#### 3.2.2. Effect of GR dose

**Fig. 7:**
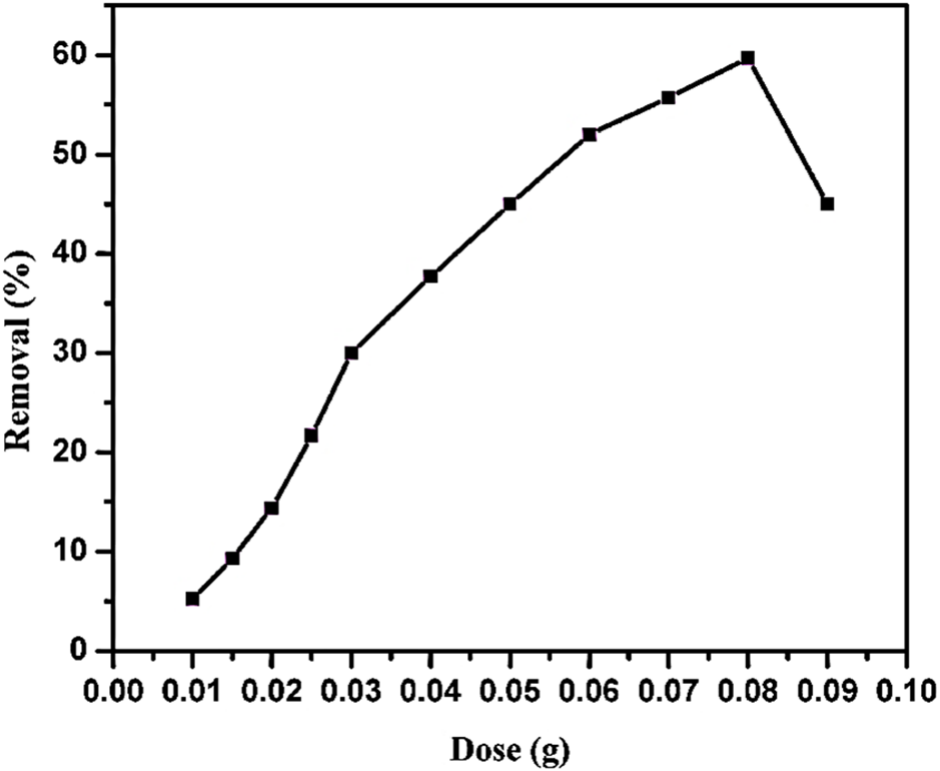
Effect of the GR doses on the 150 mg L^−1^ of MO adsorption at 25 °C and pH 7.0 in 25 mL solution.

Fig. 7 displays the effect of the GR dosage on the removal percentage of MO adsorption at pH 7.0 and 25 °C, revealing a non-linear relationship. Initially, as the GR dose increases from 0.01 g to 0.08 g, the MO removal efficiency rises steadily, indicating that a greater amount of GR provides increased active sites for MO adsorption. However, beyond this optimal dose, removal efficiency declines, likely due to the aggregation of adsorbent particles, which reduces the effective surface area and available adsorption sites. This behavior is commonly observed in adsorption studies, where an optimal adsorbent dose maximizes performance before diminishing returns occur.^35^ Based on the balance between dye removal efficiency and material consumption, a GR dose of 0.03 g is likely selected for the subsequent kinetic and effect of dye concentration studies.

#### 3.2.3. Adsorption kinetic models

**Fig. 8:**
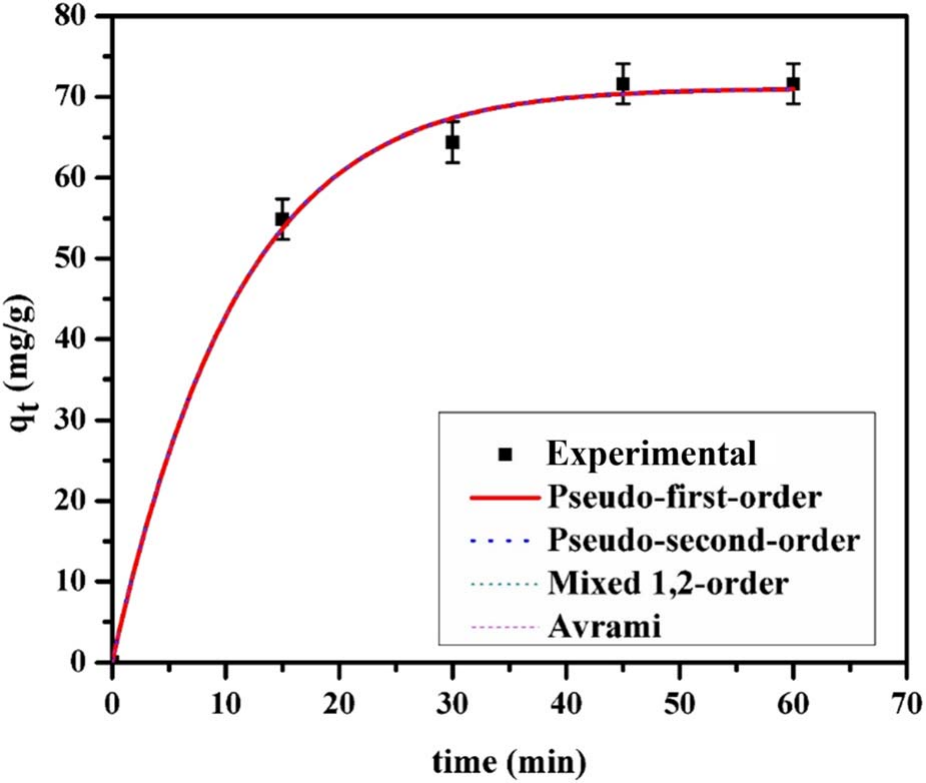
The experimental (symbols) and modeled (lines) kinetic data for 30 mg per L MO adsorption on the GR surface at 25 °C and pH 7.0.

Understanding adsorption kinetics is vital for designing efficient adsorbents, as these studies shed light on the rate at which pollutants are removed, a key aspect of optimizing the adsorption process.^52–54^ The experimental kinetic data for MO adsorption onto GR are presented in Fig. 8, alongside the fitted curves derived from four kinetic models: PFO, PSO, MO, and Avrami. The corresponding kinetic parameters, including rate constants and correlation coefficients, are summarized in Table 1. The experimental data points illustrate a rapid initial increase in adsorption capacity (*q*_*t*_), indicating a high adsorption rate at the beginning due to the abundance of available adsorption sites on the green rust. As time progresses, the adsorption rate gradually declines, suggesting that these active sites become progressively occupied, thereby reducing the capacity for further adsorption. Eventually, the *q*_*t*_ value stabilizes, reaching a plateau that signifies equilibrium. The strong alignment of the four kinetic models with the experimental adsorption data suggests a complex, multi-mechanistic process occurring on GR. The good fit of the PSO model indicates that chemisorption—likely involving strong electrostatic interactions or covalent bonding—dominates the initial rapid adsorption phase when abundant active sites are available. In contrast, the validity of the PFO model at later stages points to a transition towards diffusion-limited adsorption or weaker physisorption as surface sites become saturated. The fitting of the MO model further supports the coexistence of these mechanisms, reflecting the energetic heterogeneity of the adsorbent surface.^55^ Additionally, the agreement with the Avrami model suggests that time-dependent changes, such as nucleation or reorientation of adsorbate molecules, play a role in the adsorption process.^54^ Collectively, these findings emphasize that the adsorption mechanism is governed by a combination of factors, including surface chemistry, pore structure, and dynamic equilibria, rather than a single, idealized process.

**Table 1:** Coefficients of the kinetic models for the adsorption of MO on the GR surface^a^

Kinetics models parameters	Parameters	MO
Pseudo-first-order	*q*_e,cal_ (mg g^−1^)	71.40 ± 2.50
	*k*_1_ (min^−1^)	0.09346 ± 0.00035
	*R* ^2^	0.9973
Pseudo-second-order	*q*_e,cal_ (mg g^−1^)	81.38 ± 1.00
	*k*_2_ (g mg^−1^ min^−1^)	0.001675 ± 0.000024
	*R* ^2^	0.9988
Mixed 1,2-order	*q*_e,cal_ (mg g^−1^)	75.93 ± 2.50
	*k*_1,2_ (g mg^−1^ min^−1^)	0.02550 ± 0.00031
	*f*_2_	0.8182 ± 0.0052
	*R* ^2^	0.9988
Avrami	*q*_e,cal_ (mg g^−1^)	71.40 ± 2.50
	*k*_av_ (min^−1^)	0.06076 ± 0.00024
	*n*_av_	1.54 ± 0.50
	*R* ^2^	0.9972

a Where *t* is the time of the experiment (min), *q*_e_ is the equilibrium adsorption capacity (mg g^−1^), and *q*_*t*_ represents the adsorption capacities (mg per g adsorbent) at time *t*; *k*_1_ and *k*_2_ are the kinetic rate constant (min ^−1^); *k*_1,2_ is the adsorption rate constant (mg g^−1^ min^−1^) and *f*_2_ is the dimensionless coefficient of mixed-1,2-order; *n*_av_ is the Avrami dimensionless number and *k*_av_ is the Avrami rate constant (min^−1^).

#### 3.2.4. Adsorption isotherm

**Fig. 9:**
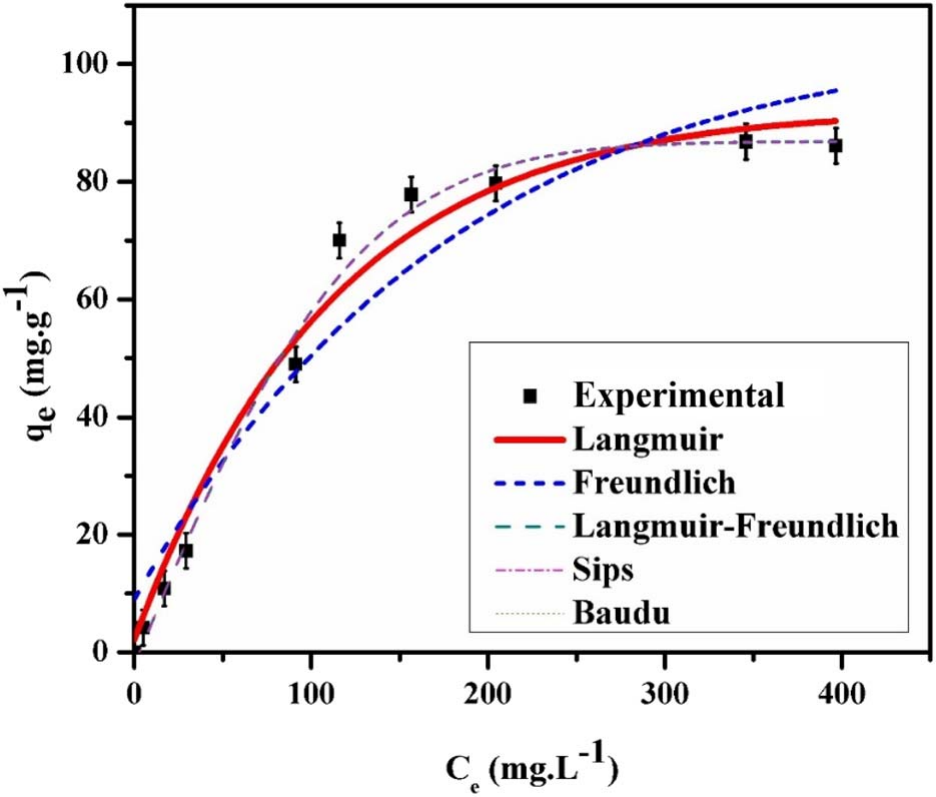
The experimental and calculated isotherm data for 25 mL MO adsorption on GR at 25 °C and pH 7.0.

The equilibrium between adsorbents and adsorbates is commonly analyzed using adsorption isotherms. The effect of MO concentrations on adsorption was examined at their respective optimal doses of GR and pH. Nonlinear fitting was applied to Langmuir, Freundlich, Langmuir–Freundlich, Baudu, and Sips isotherm models. The experimental data and fitted results are displayed in Fig. 9, with optimized parameters provided in Table 2. Findings indicated that the Langmuir–Freundlich, Baudu, and Sips models aligned most closely with the experimental data, followed by Langmuir, then Freundlich. This suggests that the GR surface contains both homogeneous and heterogeneous sites, facilitating adsorption through both monolayer and multilayer formation. The Langmuir–Freundlich and Sips models project a maximum adsorption capacity of 93.18 mg g^−1^ at pH 7.0, demonstrating the enhanced performance of nanoscale green rust in MO adsorption. Nanoscale materials generally exhibit a higher surface area, providing more binding sites and, consequently, increased adsorption capacity compared to bulk materials.

**Table 2:** Adsorption isotherm model parameters obtained from the fitting results^a^

Isotherm models	Parameters	MO (pH 7.0)
Langmuir	*Q*_m_ (mg g^−1^)	115.99 ± 2.32
	*K*_L_ (L mg^−1^)	0.0096 ± 0.0009
	*R*_L_	0.4643 ± 0.0020
	*R* ^2^	0.9666
Freundlich	*K*_F_ (L mg^−1^)	5.95 ± 0.35
	1/*n*_LF_	0.4679 ± 0.0031
	*R* ^2^	0.8951
Langmuir–Freundlich	*Q*_m_ (mg g^−1^)	93.18 ± 2.11
	*K*_LF_ (L mg^−1^)	0.01422 ± 0.0030
	*R* ^2^	0.9869
Sips	*Q*_m_ (mg g^−1^)	93.1806 ± 2.50
	*K*_s_ (L mg^−1^)	0.00082 ± 0.00013
	1/*n*_S_	1.67 ± 0.03
	*R* ^2^	0.9872
Baudu	*Q*_m_ (mg g^−1^)	93.18 ± 2.21
	*b*_0_	0.00082 ± 0.00015
	*x*	0
	*y*	0.6707 ± 0.0071
	*R* ^2^	0.9872

a *Q*_m_ is the adsorption capacity, *K*_L_ is the Langmuir constant, *K*_F_ is the Freundlich isotherm constant; *n* is the heterogeneity factor; *K*_LF_ and *K*_s_ are the Langmuir–Freundlich equilibrium and the Sips equilibrium constant, respectively; *n*_LF_ and *n*_S_ are the exponents of the Langmuir–Freundlich and Sips models, respectively; *b*_0_ is the Baudu equilibrium constant, *x* and *y* are the Baudu parameters.

The adsorption capacity of 93.18 mg g^−1^ for MO indicates the amount of dye that can be adsorbed per gram of adsorbent material under controlled laboratory conditions. To contextualize this in real-world wastewater treatment, we must consider typical dye concentrations in industrial effluents. In textile wastewater, the concentration of dyes like methyl orange typically ranges from 50 to 200 mg L^−1^,^56^ though it can exceed 500 mg L^−1^ in certain cases, depending on the dyeing process and water usage efficiency. Since wastewater is often diluted with other streams, the final dye concentration in the effluent may be lower than the initial concentration in the dye bath. Assuming a typical MO concentration of 50 mg L^−1^ in wastewater, 1 g of the adsorbent could theoretically treat approximately 1.86 L of wastewater. For higher concentrations, such as 200 mg L^−1^, the same amount of adsorbent could treat 0.47 liters of wastewater. The adsorption capacity of 93.18 mg g^−1^ is considerable and suggests that the material is effective for dye removal, particularly at moderate dye concentrations. However, in practical applications, several factors—including competition with other pollutants, pH variations, temperature fluctuations, and flow rates—can influence the performance of the adsorbent. While the adsorption capacity is promising, the material's effectiveness in real-world wastewater treatment will depend on the specific conditions and composition of the wastewater (Table 3).

**Table 3:** Reported equilibrium adsorption capacities of MO

Adsorbents	*Q*_m_ (mg g^−1^)	Ref.
Calix arene-modified lead sulphide	3.759	57
Modified clay	15.58	58
Nickel ferrite by divalent metal ions co-doping	22.24	59
Titanium dioxide	14.65	60
Activated carbon with an artificial neural network optimization modeling	7.57	61
Commercial activated carbon	113	62
AC from orange peels	33	55
CHS	7	63
M-CS/γ-Fe_2_O_3_/MWCNTs	32.78	64
Chitosan/kaolin/γ-Fe_2_O_3_	36.67	65
Protonated cross linked chitosan	89.30	66
GR	93.18	This study

#### 3.2.5. Adsorbent regeneration

**Fig. 10:**
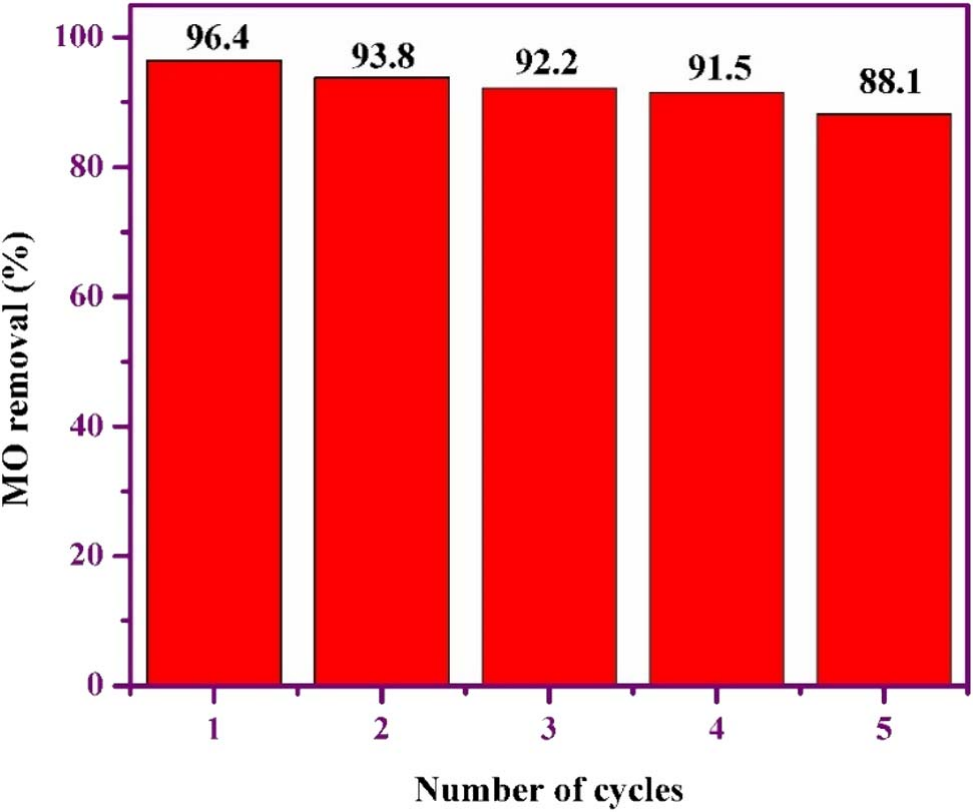
Regeneration of GR adsorbent towards MO.

Reusing adsorbents significantly lowers the overall cost of adsorption processes, as the adsorbent plays a crucial role in their efficiency. This method enhances sustainability by reducing waste and conserving resources. The adsorption–desorption cycles of MO on GR were examined over five cycles, using ethanol as the desorption solvent. As shown in Fig. 10, the removal efficiencies were 96.4%, 93.8%, 92.2%, 91.5%, and 88.1%, respectively. Although a slight decline in removal efficiency was observed with each cycle, the values remained relatively high, indicating that GR retains substantial adsorption capacity even after multiple uses. This suggests that GR could be a viable option for wastewater treatment applications.

### 3.3. Molecular simulations

#### 3.3.1. MC simulation

To explore the adsorption mechanism of MO on GR, MC simulations were performed using three distinct dry systems. These simulations provided insights into the interactions governing dye adsorption on GR. In the first system, a single MO molecule was modeled on the GR surface to analyze the specific interactions between the dye's functional groups and the active sites of GR. The second system introduced ten MO molecules to simulate monolayer formation, highlighting the competition for adsorption sites and the organization of dye molecules into a single layer on the GR surface. In the third system, twenty MO molecules were used to study the formation of multiple adsorption layers, emphasizing the interplay between dye molecules (adsorbate–adsorbate interactions) and their interactions with the GR surface (adsorbate–adsorbent interactions).

**Fig. 11:**
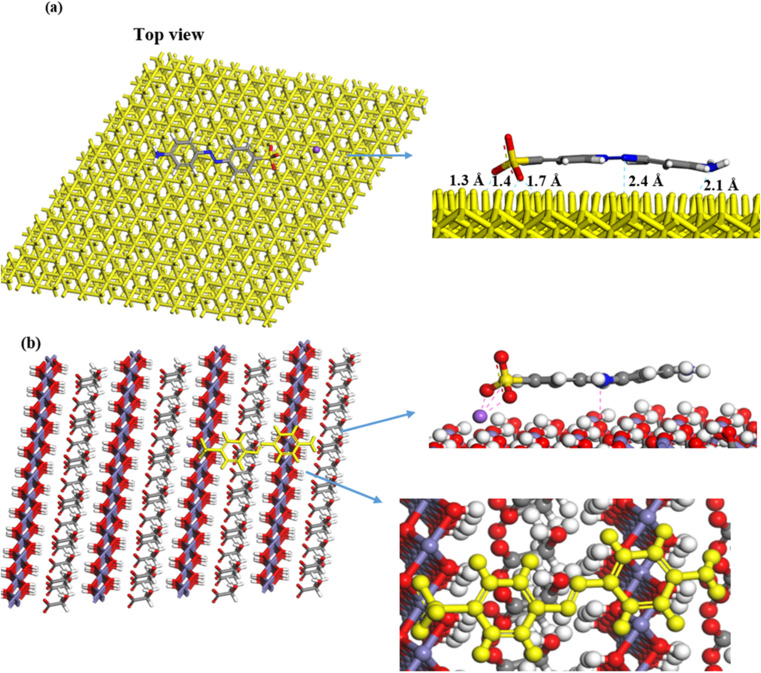
The lowest-energy configuration of the adsorption of a single MO molecule on GR (a) 001 surface and (b) 010 surface.

**Fig. 12:**
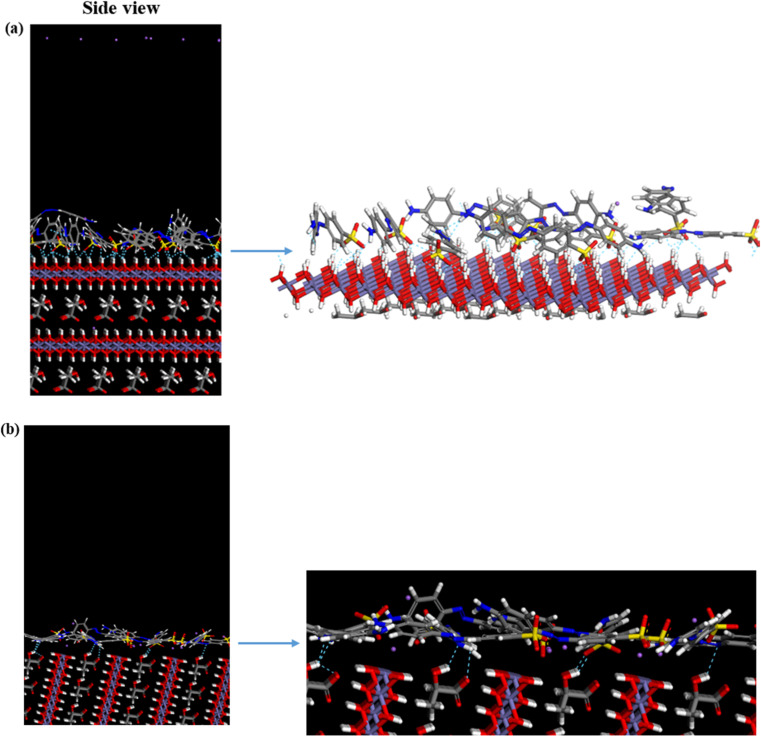
The adsorption of a single layer (10 molecules) of MO on the surface of GR (a) 001 surface and (b) 010 surface.

**Fig. 13:**
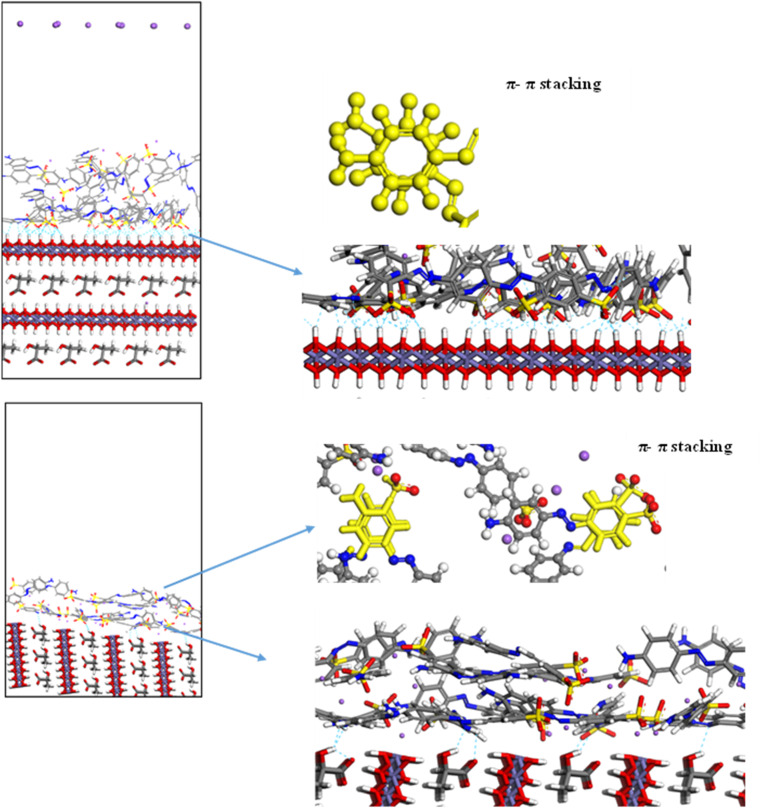
The adsorption of multilayer (20 molecules) of MO on the surface of GR (top) 001 surface and (bottom) 010 surface.

Fig. 11 illustrates the interaction of a single MO molecule with two GR surfaces. In the (001) system, Fig. 11(a) shows hydrogen bonding between the oxygen and nitrogen atoms of MO and hydrogen atoms in GR, with bond lengths ranging from 1.3 Å to 2.4 Å. In the (010) system, Fig. 11(b) highlights electrostatic interactions, with close-contact distances ranging from 2.0 Å to 3.3 Å. The Fe center in GR likely interacts with the π-electrons of the aromatic MO ring, a type of interaction that is significant in many adsorption processes. For the monolayer simulation in the (001) system, Fig. 12(a) shows 10 MO molecules adsorbed onto the GR surface, with hydrogen bond lengths ranging from 1.3 Å to 2.4 Å. In the (010) system, Fig. 12(b) shows hydrogen bond lengths ranging from 1.47 Å to 2.46 Å. In the multilayer simulation for the (001) system, Fig. 13(a) shows the GR surface covered with 20 MO molecules, with hydrogen bond lengths ranging from 1.3 Å to 2.5 Å. For the (010) system, Fig. 13(b) displays hydrogen bond lengths between 1.47 Å and 2.46 Å, with the interaction between the two layers characterized by π–π stacking interactions.

#### 3.3.2. MD simulation

**Fig. 14:**
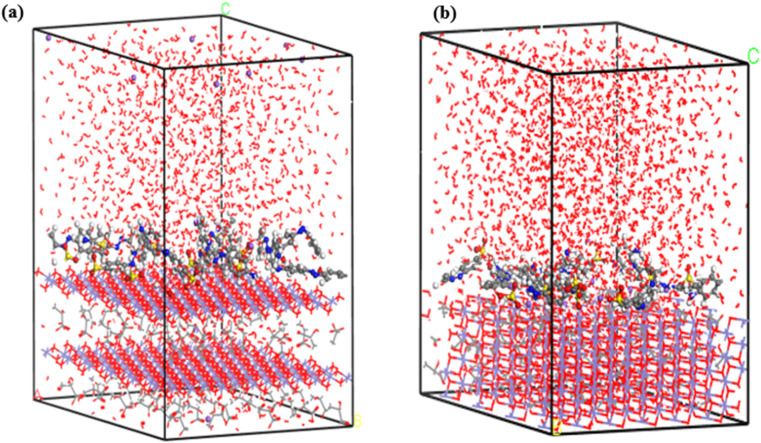
The final MD snapshots of the adsorption of a single layer (10 molecules) in aqueous solution of MO on the surface of GR (a) 001 surface and (b) 010 surface.

**Fig. 15:**
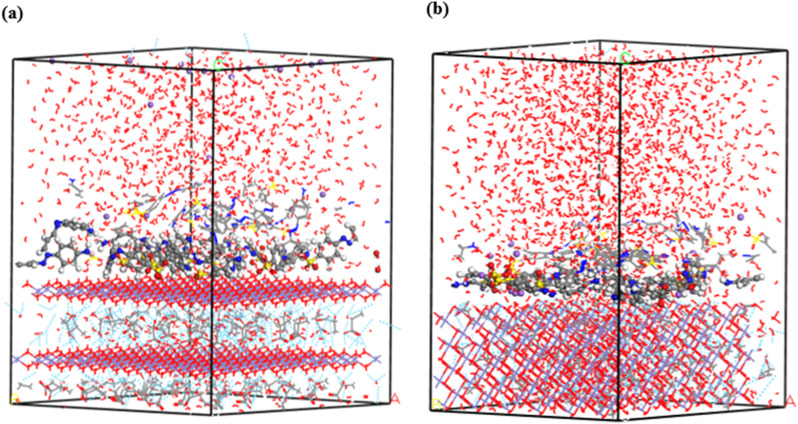
The final MD snapshots of the adsorption of multilayer (20 molecules) in aqueous solution of MO on the surface of GR (a) 001 surface and (b) 010 surface.

Water molecules were incorporated into the single- and multilayer systems derived from the initial MC simulations to more accurately replicate experimental conditions and simulate the environment in which dye molecules are adsorbed. MD simulations were subsequently performed on these hydrated systems at 25 °C to investigate whether the dyes would preferentially adsorb onto the GR surface or desorb and disperse into the surrounding water. Including water molecules enabled the simulation to account for competition between dye and water molecules for adsorption sites on the GR surface. The final configurations from the MD simulations, presented in Fig. 14 and 15, confirm that the MO molecules remained adsorbed on the GR surface. This outcome indicates strong interactions between the dye molecules and the active sites on the rust surface, which persisted even in the presence of water. These findings align with experimental results, which suggest a high adsorption capacity of dyes on the GR surface.

### 3.4. Adsorption and photocatalysis for MO and MB removal

**Fig. 16:**
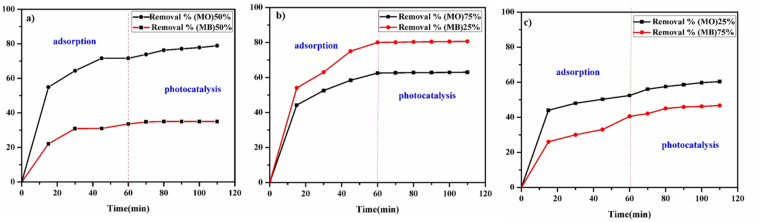
Adsorption of mixed MO and MB for 60 minutes, followed by photocatalysis for 50 minutes, at different dye concentrations: (a) 50% MO, 50% MB; (b) 75% MO, 25% MB; (c) 25% MO, 75% MB, at pH 7.

**Fig. 17:**
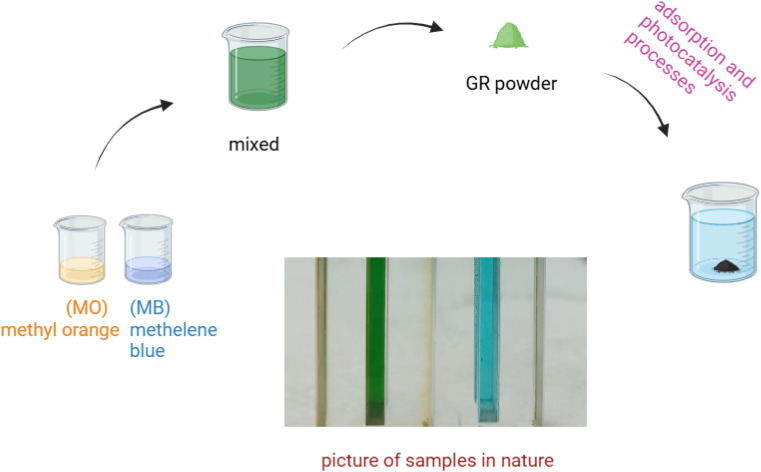
Removing mixed of anionic dye (MO 12.5 mL at 30 mg L^−1^) and cationic dye (MB, 12.5 mL at 30 mg L^−1^) at pH 7.0 and 25 °C.

The GR alone is ineffective in removing cationic dyes such as MB. Interestingly, the presence of MO appeared to enhance MB removal, suggesting that MO may play a role in the transport or interaction mechanism of MB with GR (Fig. 16). In the photocatalysis experiments (Fig. 17), the continued presence of MO further influenced MB removal efficiency over time. The photocatalysis process removed both dyes at a relatively low rate but enhanced overall degradation efficiency by facilitating additional breakdown pathways. LDHs can act as photocatalysts for pollutant degradation, including dyes and organic contaminants.^67^ Their photocatalytic activity is primarily due to their layered structure, metal composition, and tunable bandgap. LDHs, particularly those incorporating transition metals (*e.g.*, Fe, Zn, Ti, Co, Ni, Mn, Cu), are capable of absorbing UV or visible light. Upon irradiation, electrons (e^−^) are excited from the valence band (VB) to the conduction band (CB), generating holes (h^+^) in the VB:LDH + *hν* → e_(CB)_^−^ + h_(VB)_^+^

The photogenerated electrons (e^−^) and holes (h^+^) actively participate in redox reactions. The excited electrons (e^−^) reduce oxygen molecules, leading to the formation of superoxide radicals (˙O_2_^−^):O_2_ + e^−^ → ˙O_2_^−^

Holes (h^+^) oxidize water molecules or hydroxide ions (OH^−^) to generate hydroxyl radicals (˙OH):H_2_O + h^+^ → ˙OH + H^+^OH^−^ + h^+^ → ˙OH

Reactive oxygen species (ROS), including hydroxyl radicals (˙OH) and superoxide radicals (˙O_2_^−^), degrade dye molecules into smaller, non-toxic byproducts.MO + ˙OH → decomposed products + CO_2_ + H_2_O

Direct oxidation by holes (h^+^) can also contribute to dye degradation.Dye + h^+^ → oxidized products

## 4. Conclusion

Green rust was prepared using unscalable, eco-friendly method, using iron-containing precursor as an abundant, sustainable raw material. The prepared green rust was used as an adsorbent and photocatalyst for methylene blue and methyl orange removal. The influence of adsorbent dosage and adsorption isotherms, as well as kinetics, was studied under optimal pH conditions. The sample showed the highest adsorption capacity of 93.18 mg MO per g GR at pH 7.0. The effect of initial dye concentration was also evaluated and analyzed using various kinetic models, the pseudo-second-order and mixed 1,2-order models demonstrated significantly better fits compared to the pseudo-first-order and Avrami models. To further elucidate the adsorption mechanism, Monte Carlo simulations were employed, revealing that the primary interactions driving dye removal involve the functional groups of the dye and the active sites on GR. MD simulations confirmed that the dye molecules remain adsorbed on the GR surface in aqueous conditions at 25 °C. This study highlights the potential of GR as a sustainable and abundant material for effective dye removal.

## Data availability

All data generated or analysed during this study are included in submitted manuscript.

## Conflicts of interest

There are no conflicts to declare.

## Supplementary Material

RA-015-D5RA01508A-s001
