# Supplementary material for: Methylene blue and methyl orange removal using green rust as a low-cost, sustainable adsorbent and photocatalyst
Source: RSC Adv. 2025 Jun 2;15(23):18403–18. doi: 10.1039/d5ra01508a (PMC12129064; doi:10.1039/d5ra01508a)
Supplement: RA-015-D5RA01508A-s001 [file RA-015-D5RA01508A-s001.pdf]

Supplementary Information (SI) for RSC Advances.
This journal is © The Royal Society of Chemistry 2025

# Supplementary Information

**Isothermal models**

The Freundlich equation :

$$Q_e = K_f C_e^{1/n} \qquad (1)$$

where $K_f$ is a Freundlich isotherm constant, $C_e$ is an equilibrium dye concentration (mg/l) and 1\n represents the strength of adsorption.

The Langmuir isotherm:

$$Q_e = \frac{Q_m K_L C_e}{1 + K_L C_e} \qquad (2)$$

where $Q_e$ is the amount of dye adsorbed at equilibrium (mg/g), $K_L$ is the Langmuir adsorption constant (mg/g), $Q_m$ is the monolayer adsorption (mg/g) and $C_e$ is the equilibrium dye concentration (mg/L). The separation factor, known as RL, determines the degree of adhesion between an adsorbent and an adsorbate and is calculated from

$$RL = \frac{1}{1 + K_L C_0} \qquad (3)$$

where $C_o$ is the initial dye concentration. A higher RL value (closer to 1) indicates a stronger, favorable attraction, while lower values (closer to 0) indicate a weaker, unfavorable interaction.

Langmuir-Freundlich isotherm:

$$Q_e = \frac{Q_m (K_{LF} C_e)^n}{1 + (K_{LF} C_e)^n} \qquad (4)$$

Sipes isotherm is a flexible model that combines also Langmuir and Freundlich, allowing for diverse scenarios like heterogeneous or homogeneous, monolayer or multilayer, and predicts saturation.[31].

$$Q_e = \frac{Q_m K_S C_e^{1/n_s}}{1 + K_S C_e^{1/n_s}} \quad (5)$$

where $K_S$ is the Sipes equilibrium constant

The Baudu isotherm model:

$$Q_e = \frac{Q_m b_0 C_e^{(1+x+y)}}{1 + b_0 C_e^{(1-x)}} \quad (6)$$

where $Q_m$ is the Baudu maximum adsorption capacity (mg/g), $b_0$ is the equilibrium constant, *x* and *y* are the Baudu parameters.

**Kinetics models**

The Pseudo-first-order model (PFO):

$$q_t = q_e\,(1 - e^{(-k_1 t)}) \quad (7)$$

Where $k_1$ is the kinetic rate constant (min $^{-1}$),t is the time of the experiment (min), $q_e$ is the equilibrium adsorption capacity (mg adsorbate/g adsorbent) and $q_t$ represents the adsorption capacities (mg adsorbate / g adsorbent) at time t.

Pseudo-second-order model (PSO):

$$q_t = \frac{k_2 q_e^2 t}{1 + k_2 q_e t} \quad (8)$$

Where $k_2$ is the kinetic rate constant (min $^{-1}$).

Mixed-1,2-order model (MO):

$$q_t = q_e \frac{1 - e^{(-kt)}}{1 - f_2 e^{(-kt)}} \quad (9)$$

Here, k is the adsorption rate constant ($mg.g^{-1}.min^{-1}$)and $f_2$ is the dimensionless coefficient of mixed-1,2-order.

Avrami model:

$$q_t = q_e(1 - e^{(-k_{av}t)^{n_{av}}}) \quad (10)$$

Where $n_{av}$ is the Avrami dimensionless number and $k_{av}$ is the Avrami rate constant ($min^{-1}$).
